# Synthetic biology tools for programming gene expression without nutritional perturbations in *Saccharomyces cerevisiae*

**DOI:** 10.1093/nar/gkt1402

**Published:** 2014-01-20

**Authors:** R. Scott McIsaac, Patrick A. Gibney, Sunil S. Chandran, Kirsten R. Benjamin, David Botstein

**Affiliations:** ^1^The Lewis-Sigler Institute for Integrative Genomics, Princeton University, Princeton, NJ 08544, USA, ^2^Amyris, Inc., Emeryville, CA 94608, USA, ^3^Division of Chemistry and Chemical Engineering, California Institute of Technology, Pasadena, CA 91125, USA and ^4^Department of Molecular Biology, Princeton University, Princeton, NJ 08544, USA

## Abstract

A conditional gene expression system that is fast-acting, is tunable and achieves single-gene specificity was recently developed for yeast. A gene placed directly downstream of a modified *GAL1* promoter containing six Zif268 binding sequences (with single nucleotide spacing) was shown to be selectively inducible in the presence of β-estradiol, so long as cells express the artificial transcription factor, Z_3_EV (a fusion of the Zif268 DNA binding domain, the ligand binding domain of the human estrogen receptor and viral protein 16). We show the strength of Z_3_EV-responsive promoters can be modified using straightforward design principles. By moving Zif268 binding sites toward the transcription start site, expression output can be nearly doubled. Despite the reported requirement of estrogen receptor dimerization for hormone-dependent activation, a single binding site suffices for target gene activation. Target gene expression levels correlate with promoter binding site copy number and we engineer a set of inducible promoter chassis with different input–output characteristics. Finally, the coupling between inducer identity and gene activation is flexible: the ligand specificity of Z_3_EV can be re-programmed to respond to a non-hormone small molecule with only five amino acid substitutions in the human estrogen receptor domain, which may prove useful for industrial applications.

## INTRODUCTION

Numerous strategies have emerged for controlling levels of gene expression in yeast. These include introducing sequences that disfavor nucleosome localization near the transcription start site (1), varying both the affinity and number of binding sites for transcriptional activators in the promoter (2–4) and randomization of core promoter elements (5). Protein levels can also be greatly influenced by the 5′-UTR and 3′-UTR sequences. The sequence ‘AAA’ at positions −1 → −3 of the gene’s initial ATG favor high protein expression, while out-of-frame AUGs in the 5′-UTR near the start codon reduce protein levels (6). In the 3′-UTR, higher A/T content upstream of the polyadenylation site correlates with higher protein expression (7). While promoter strength and protein expression can be tuned in a variety of ways, it is also desirable to selectively introduce or remove selected gene products so that the effects of gain or loss of function can be assayed (3,8–10).

Conditional induction of gene expression has relied heavily on placing genes under the control of nutrient-responsive DNA sequences (11,12). Promoters responsive to the tetracycline analog doxycycline and to hormones such as β-estradiol, both of which are inert with respect physiology, have also been developed (13–15). In the case of β-estradiol, artificial transcription factors (ATFs) containing a DNA binding domain, a hormone binding domain and an activation domain are expressed in the cell. The ATF Gal4dbd.hER.VP16 (GEV) is a fusion of the *Saccharomyces cerevisiae* Gal4p DNA binding domain, the ligand binding domain of the human estrogen receptor (hER) and the activation domain of viral protein 16 (VP16, from herpes simplex virus) (14,15). In the absence of inducer, GEV is sequestered to the cytoplasm where, through the hER, it interacts with the Hsp90 chaperone complex (15,16). On addition of inducer, GEV dissociates from the Hsp90 chaperone complex, localizes to the nucleus and activates expression of genes placed downstream of promoters containing Gal4p-responsive DNA elements (14,15).

The GEV system has many desirable properties. It is fast-acting, has a graded input–output relationship with respect to inducer concentration, activates expression in all cells and works in diverse media formulations (including those using 2% glucose as a carbon source) (15,17). However, the presence of the Gal4p DNA binding domain results in unwanted activation of many off-target genes (as well as indirect repression of others), resulting in defective growth at moderate-to-high levels of inducer (15). By replacing the DNA binding domain of GEV with others that have few-to-no binding sites in the genome [as detailed in (3,18)], the desirable properties of GEV can be retained while eliminating any unwanted off-target effects. One such protein, Z_3_EV, is identical to GEV, except for the DNA binding domain, which is replaced with Zif268 (Z_3_ refers to the fact that Zif268 has three zinc fingers, which results in 9 bp of DNA specificity).

In this manuscript, we tested promoter design principles and expanded the range of Z_3_EV functionality. Specifically, the effects of 5′-UTR sequence, binding site copy number and binding site spacing on gene expression were tested in Z_3_EV-responsive promoters. We find that a single binding site is enough to make a promoter Z_3_EV-inducible. In promoters with multiple binding sites, inter-binding site spacing can affect the level of induction, especially at sub-saturating concentrations of inducer. Whereas previously we exclusively used *GAL1* promoters modified to include Z_3_EV binding sites, here we test the ability of other promoter chassis to be made inducible by Z_3_EV with a high dynamic range. Finally, we show that changing five amino acids in the hER domain is sufficient to make Z_3_EV completely non-responsive to β-estradiol, but strongly responsive to a non-steroid small molecule.

## MATERIALS AND METHODS

### Strains, media and cloning

The parent strain used in the promoter- and ATF-characterization experiments is a derivative of CEN.PK113-7 d (19) in which *URA3* was replaced by NatMX. NatMX is flanked by targeting sequences for the endonuclease F-CphI to facilitate its removal if desired. Chemostat and growth experiments were performed using DBY12000, an s288c-derivative with a functional *HAP1* allele (8). For batch experiments, yeast cells were cultured in either complete synthetic medium lacking uracil (CSM-U; 2% dextrose) or yeast extract-peptone-dextrose medium (1% yeast extract, 2% bactopeptone and 2% dextrose).

Transformations were performed using a standard lithium acetate method (20). All cloning was performed using DNA gap repair in the yeast strain CEN.PK2-1c, a tryptophan auxotroph (*MATa; ura3-52; trp1-289; leu2-3,112; his3Δ 1; MAL2-8^C^; SUC2*). Complete synthetic medium lacking tryptophan (CSM-W; 2% dextrose) was used as selective medium for yeast outgrowth following transformation. Yeast transformations were miniprepped directly from liquid cultures and transformed into XL1-Blue cells (Agilent). Single colonies were selected on LB-CARB (0.5% yeast extract, 1% tryptone, 0.5% NaCl and 0.1 g/l carbenicillin) and proper assemblies were confirmed via restriction digestion and Sanger sequencing as described previously (21). The vector used for assembly is a derivative of the *TRP1*-marked yeast shuttle vector pRS414 with the LacZ open reading frame disrupted by a pair of linkers, referred to as 0 and 9, between which DNA assemblies were constructed: Linker 0 = GACGGCACGGCCACGCGTTTAAACCGCC; Linker 9 = CGGTGTTTAAACCCCAGCGCCTGGCGGG. Genomic insertions were generated via transformation of two linear DNA fragments with overlap in the *URA3* marker and subsequent selection for growth on CSM-U agar medium. Colony polymerase chain reaction was used to confirm proper insertion at the *BUD9* locus.

β-estradiol (Sigma-Aldrich) and 4′-4′-dihydroxybenzyl (DHB; Austin Chemical Company) were dissolved in 100% ethanol at 25 mM and stored at 4°C. Synthetic promoters and yeast codon-optimized ATFs were synthesized commercially (SGI-DNA, La Jolla, CA, USA). Codon-optimized sequences were determined using Integrated DNA Technologies’ web interface (Coralville, IA). The fluorescent reporter, GFP-Dasher, was obtained from DNA2.0 (Menlo Park, CA, USA). Restriction enzymes were obtained from New England Biolabs (Ipswich, MA, USA).

### Flow cytometry

For dose–response experiments, saturated overnight cultures in CSM-U medium were diluted 1:200 in 5 ml of fresh CSM-U medium. Cells were incubated with different levels of β-estradiol or DHB for ∼12 h. Approximately 10^7^ cells were harvested and re-suspended in phosphate buffered saline supplemented with 0.1% Tween-20. Green fluorescent protein (GFP) fluorescence measurements were made with a BD FACSAria II (BD Biosciences, Sparks, MD, USA). Mean fluorescence values were obtained from a minimum of 10 000 cells in each sample, and the fluorescence detector calibration settings maintained constant between experiments. Positive and negative GFP controls were used to ensure that the instrument maintained proper calibration and to ensure proper normalization. For kinetic experiments, cells were allowed to grow for 4 h before introduction of inducer. For all flow cytometry experiments, data analysis was performed in MATLAB, as described previously (18).

### Chemostat experiment

Yeast cells were cultured in a 500-ml chemostat (Sixfors, Infors AG, Bottmingen, Switzerland) under phosphate limitation (20 mg/l). The culture was grown at 30 °C, stirred at 400 rpm, aerated with filtered humidified air, and maintained at a volume of 300 ml. The growth rate was maintained at 0.17 h^−1^ (culture doubling time = 4.08 h). Batch growth was initiated from a 1:60 dilution of an overnight culture (also grown in phosphate-limited chemostat medium). Cells were grown to steady state (as determined by optical density and cell size) before addition of DHB.

### Microarrays

RNA extraction, labeling and hybridization were performed as described previously (22) with slight modifications. Briefly, chemostat samples (∼5 ml) were vacuum-filtered onto 0.45 -µm nylon membranes (Millipore, HNWP02500), placed in 2-ml locking lid tubes (FisherBrand, 02-681-291) and flash-frozen in liquid nitrogen. Crude RNA was extracted with a standard acid–phenol procedure and cleaned with RNeasy mini columns (QIAGEN, Valencia, CA, USA) before mRNA amplification and labeling with the Agilent Quick-Amp Labeling Kit (Part No. 5190-0424). Amplification and labeling were performed according to the manufacturer’s instructions with half the volume of each reagent and 0.6 µl of Cy3 or Cy5 dye. Reference RNA was extracted from cells harvested just before addition of DHB. Agilent Yeast Oligo V2 microarrays (8 × 15 k) were hybridized for 17 h at 65°C on a rotisserie at 20 rpm. Following a series of wash steps, hybridized microarrays were scanned and raw data were extracted with Agilent Feature Extractor Software version 9.5.

## RESULTS

**Figure 1.**
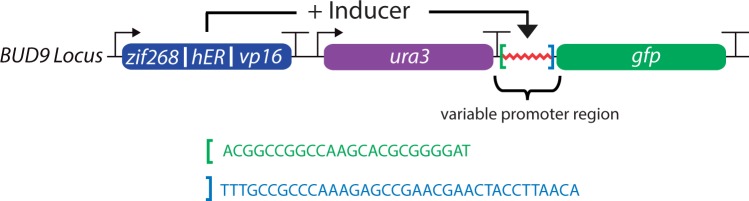
Schematic of *BUD9* locus in engineered yeast strains. Universal primer sites (shown in green and blue) flank the variable promoter region in all strains. Gene terminators are represented by ‘T’ in the figure. The *ENO2* terminator follows Z_3_EV and the *GAL80* terminator follows GFP. The promoter/terminator pair surrounding *URA3* is the same as the native gene.

For all promoter characterization experiments, a contiguous string of DNA ‘parts’ replaces the entire *BUD9* open reading frame. Z_3_EV is constitutively expressed from the *ACT1* promoter, followed sequentially by *URA3* (used as a selection marker for genomic integration), the synthetic promoter to be tested and GFP (Figure 1). The level of gene expression from each promoter was measured from three independent cultures (from three independent colonies) via flow cytometry in the absence or presence (medium level = 10 nM or high level = 1 µM) of β-estradiol. A complete list of strains can be found in Table 1, and the sequences of DNA parts can be found in the Supplement. In addition to the figures that illustrate binding site location and spacing, each promoter is assigned a unique number (P1–P15).

**Table 1.** List of yeast strains used in experiments

Strain	Genotype	Source
DBY12000	*GAL2^+^* s288c with repaired *HAP1* allele	(8)
DBY19000	*ura3Δ*::NatMX	This study
DBY19004	*ura3Δ*::NatMX, *bud9*Δ::ACT1pr-Z_3_EV-ENO2term-*URA3*-Z_3_EVpr(P1)-GFP-Gal80term	This study
DBY19017	*ura3Δ*::NatMX, *bud9*Δ::ACT1pr-Z_3_EV-ENO2term-*URA3*-CM2pr(P15)-GFP-Gal80term	This study
DBY19018	*ura3Δ*::NatMX, *bud9*Δ::ACT1pr-Z_3_EV-ENO2term-*URA3*-HOP2pr(P12)-GFP-Gal80term	This study
DBY19019	*ura3Δ*::NatMX, *bud9*Δ::ACT1pr-Z_3_EV-ENO2term-*URA3*-HOP1pr(P11)-GFP-Gal80term	This study
DBY19050	*ura3Δ*::NatMX, *bud9*Δ::ACT1pr-Z_3_EV-ENO2term-*URA3*-CM1pr(P14)-GFP-Gal80term	This study
DBY19052	*ura3Δ*::NatMX, *bud9*Δ::ACT1pr-Z_3_EV-ENO2term-*URA3*-Z_3_EVpr(P2)-GFP-Gal80term	This study
DBY19053	*ura3Δ*::NatMX, *bud9*Δ::ACT1pr-Z_3_EV-ENO2term-*URA3*-Z_3_EVpr(P3)-GFP-Gal80term	This study
DBY19054	*ura3Δ*::NatMX, *bud9*Δ::ACT1pr-Z_3_EV-ENO2term-*URA3*-Z_3_EVpr(P4)-GFP-Gal80term	This study
DBY19055	*ura3Δ*::NatMX, *bud9*Δ::ACT1pr-Z_3_EV-ENO2term-*URA3*-Z_3_EVpr(P6)-GFP-Gal80term	This study
DBY19056	*ura3Δ*::NatMX, *bud9*Δ::ACT1pr-Z_3_EV-ENO2term-*URA3*-Z_3_EVpr(P5)-GFP-Gal80term	This study
DBY19058	*ura3Δ*::NatMX, *bud9*Δ::ACT1pr-Z_3_EV-ENO2term-*URA3*-CrippledCYC1pr(P7)-GFP-Gal80term	This study
DBY19059	*ura3Δ*::NatMX, *bud9*Δ::ACT1pr-Z_3_EV-ENO2term-*URA3*-CrippledCYC1pr(P8)-GFP-Gal80term	This study
DBY19061	*ura3Δ*::NatMX, *bud9*Δ::ACT1pr-Z_3_EV-ENO2term-*URA3*-CrippledCYC1pr(P9)-GFP-Gal80term	This study
DBY19063	*ura3Δ*::NatMX, *bud9*Δ::ACT1pr-Z_3_EV-ENO2term-*URA3*-CrippledCYC1pr(P10)-GFP-Gal80term	This study
DBY19065	*ura3Δ*::NatMX, *bud9*Δ::ACT1pr-Z_3_EV-ENO2term-*URA3*-DAN1pr(P13)-GFP-Gal80term	This study
DBY19068	*ura3Δ*::NatMX, *bud9*Δ::CYC1pr-Z_3_E(4S)V-YPR052Cterm -*URA3*-Z_3_EVpr(P1)-GFP-Gal80term	This study
DBY19070	*ura3Δ*::NatMX, *bud9*Δ::ACT1pr-Z_3_E(4S)V-YPR052Cterm-*URA3*-Z_3_EVpr(P1)-GFP-Gal80term	This study

All 19 000 series yeast strains are in the CEN-PK113-7d background. In the genotypes, ‘pr’ indicates a promoter and ‘term’ indicates a terminator. Z_3_EVpr indicates a modified version of the *GAL1* promoter that contains Zif268 binding sites. All Z_3_EV-responsive promoters have a unique number (P1, P2, … , P15).

### Modified *GAL1* promoters

**Figure 2.**
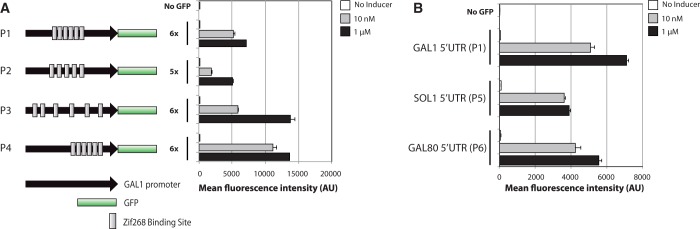
Engineered *GAL1* promoter variants. (**A**) Measuring the effects of Zif268 binding site location and spacing on Z_3_EV-mediated gene induction. Schematic cartoons (not drawn to scale) illustrate the tested promoter architectures and bar plots are measurements of GFP intensity in the presence or absence of inducer following ∼12 h of induction. (**B**) Measuring the effects of 5′-UTR sequence on Z_3_EV-mediated gene induction, by replacing the *GAL1* 5′-UTR with that of *SOL1* or *GAL80*. Error bars represent the standard deviation of fluorescence from three independent colonies/cultures.

Previously, we removed the three canonical Gal4p activation sequences in the *GAL1* promoter and replaced them with six Zif268 binding sites with single nucleotide spacing (Figure 2A, P1) (3). A modified *GAL1* promoter in which five Zif628 binding sites have 9-bp spacing remains inducible at both 10 nM and 1 µM β-estradiol (Figure 2A, P2), demonstrating that there is some flexibility over the exact choice of binding site spacing.

In promoters containing six randomly dispersed binding sites (Figure 2A, P3), or six binding sites with single nucleotide spacing (Figure 2A, P4), expression in the presence of 1 µM β-estradiol nearly doubled with respect to our original promoter design, P1. In both P3 and P4, the binding site closest to the start codon is 235 bp and 242 bp, respectively (∼100 bp upstream of the transcription start state [TSS]). In P1 and P2, the binding site closest to the start codon is 408 and 385 bp (∼250 bp upstream of the TSS), respectively. These results demonstrate that moving binding sites closer to the TSS of modified *GAL1* promoters can increase induced expression without increasing basal expression (i.e. in the absence of inducer, the distribution of fluorescence intensities is indistinguishable from cells lacking GFP), leading to an overall increase in the system’s dynamic range.

To test the effect of 5′-UTR sequence on induciblity, the *GAL1* 5′-UTR was removed and replaced with that of *GAL80* or *SOL1* (23), both of which encode more stable transcripts than *GAL1* in glucose-grown cells (24). In glucose, the average half-life of mRNAs in yeast is 20.1 ± 8.6 min (24). The *GAL80* mRNA half-life is 27 min and that of *SOL1* is 79 min, whereas the half-life of *GAL1* mRNA is 19 min (24). Despite *GAL80* and *SOL1* being more stable transcripts, we find their 5′-UTR sequence to be slightly deleterious to overall protein production in the context of the modified *GAL1* promoter (Figure 2B, P5 and P6).

### Altering promoter context and binding site copy number

**Figure 3.**
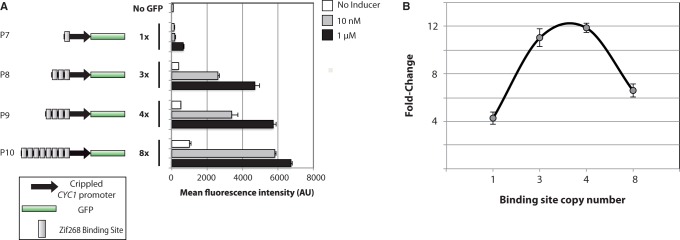
The effect of Zif268 binding site copy number on expression in crippled *CYC1* promoters. (**A**) Measuring the effect of Zif268 binding site copy number on basal and induced expression when placed directly upstream of the crippled *CYC1* promoter from (25). (**B**) The fold-change of expression at 1 µM β-estradiol over uninduced cells as a function of binding site copy number. Error bars represent the standard deviation of fluorescence from three independent colonies/cultures.

Different numbers of Zif268 binding sites were placed upstream of a 249-bp crippled *CYC1* promoter sequence from (25). From these experiments, we find that that at both 10 nM and 1 µM β-estradiol, induction correlates with binding site copy number (Figure 3A). One binding site is sufficient to make the promoter inducible (Figure 3A, P7). Unlike in the case of the modified *GAL1* promoters, increased induction comes at the cost of increased basal expression in the absence of inducer (the basal expression from the promoter with eight binding sites is ∼6-fold greater than that with a single binding site). While going from 4 to 8 binding sites results in a 17% increase in GFP expression at 1 µM β-estradiol, the basal expression doubles. This results in the fold-change of induction being non-monotonic with respect to binding site copy number (Figure 3B).

### Increasing the number of promoter chassis

**Figure 4.**
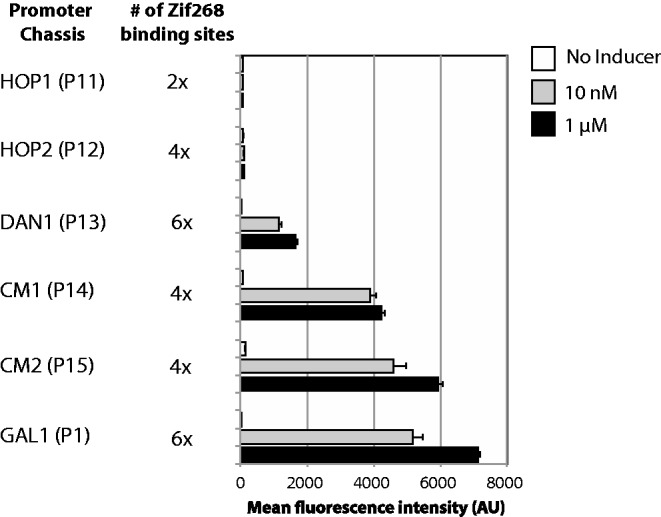
Testing the ability of alternative promoter chassis to be made inducible. Data are shown for six different promoters, including our initial modified *GAL1* promoter design (P1). CM1 and CM2 are two different variants of ‘*CYC1 Minimal*’ promoters we designed to remove basal activity from the wild-type *CYC1* promoter. Error bars represent the standard deviation of fluorescence from three independent colonies/cultures.

Based on the aforementioned results, it seemed reasonable that other promoters could be made inducible as well. *DAN1, HOP1* and *HOP2* are strongly repressed in asexually grown yeast. *DAN1* encodes a cell wall mannoprotein that is expressed under anaerobic conditions (26,27). *HOP1* and *HOP2* gene products are both involved in the regulation of meiosis (28,29). Whereas a *DAN1* promoter containing Zif268 binding sequences was made inducible by β-estradiol (Figure 4, P13), the modified *HOP1* and *HOP2* promoters were not (Figure 4, P11 and P12). ‘*CYC1* Minimal’ promoters (CM1 and CM2), whose expression was attenuated based on results from Guarente and colleagues (30), were also made strongly inducible by the addition of Zif268 binding sites (Figure 4, P14 and P15).

### Changing the ligand specificity of Z_3_EV

After finding that Z_3_EV-responsive promoters could be engineered in a straightforward fashion, we wanted to see if they could be made to respond to a non-hormone small molecule. It has previously been demonstrated that ligand specificity of hER can be altered through directed evolution using two-hybrid screening in yeast (31–34). Indeed, mutant estrogen receptors have been engineered with enhanced specificity for resveratrol-like compounds (33), androgens (34), and synthetic small molecules, including DHB and 2,4-di(4-hydroxyphenyl)-5-ethylthiazole (L9) (31,32). Based on results from (31), we synthesized a codon-optimized variant of Z_3_EV, called Z_3_E(4S)V, that contains five hER domain amino acid substitutions. Z_3_EV and Z_3_E(4S)V contain amino acids 282–576 of the alpha subunit of the hER. According to this numbering, Z_3_E(4S)V contains the hER substitutions L346I, A350M, M388Q, G521S and Y526D.

**Figure 5.**
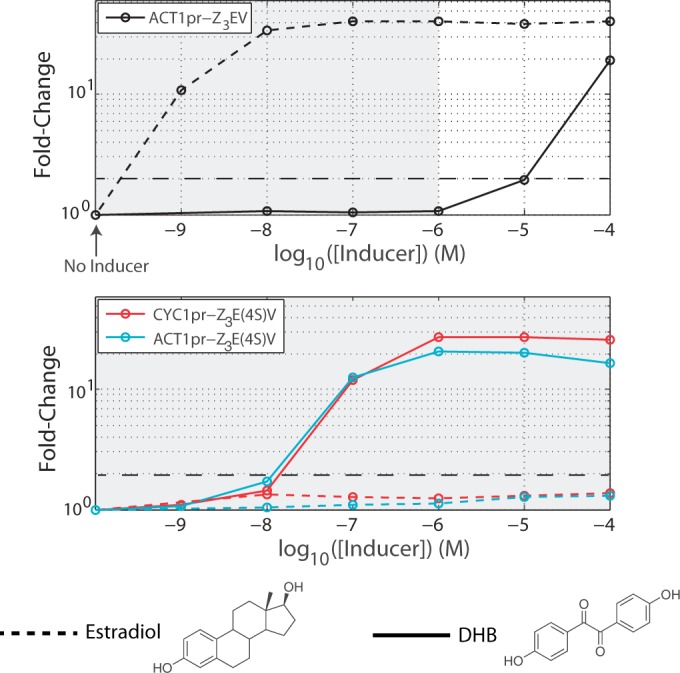
Dose–response curves for wild-type and mutant Z_3_EV variants. Responses to β-estradiol and DHB (short-dashes and solid lines, respectively) are shown for Z_3_EV (top) and Z_3_E(4S)V (bottom). A horizontal line (long dashes) indicates a 2-fold change in expression of GFP in each plot. Gray shading indicates concentrations of DHB to which Z_3_EV is not activated (top) or concentrations of β-estradiol to which Z_3_E(4S)V is not activated (bottom). Fluorescence measurements were made using flow cytometry.

**Figure 6.**
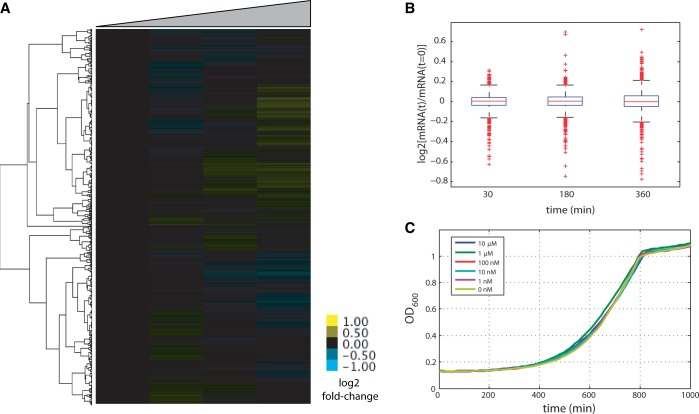
DHB has no effect on gene expression or growth. 1 µM DHB was introduced to phosphate-limited chemostat culture of strain DBY12000 at steady state. Samples were harvested at t = 0, 30, 180 and 360 min following DHB addition. (**A**) Hierarchical clustering of global gene expression response to DHB. Data are normalized to the t = 0 sample. (**B**) Data from (A) represented as a box plot. (**C**) DBY12000 grown in the presence of different amounts of DHB.

We find that the wild-type Z_3_EV construct is non-responsive with respect to the small molecule DHB at or below concentrations of 1 µM (Figure 5). Z_3_E(4S)V is induced by 100 nM DHB, but is non-responsive to any of the tested concentrations of β-estradiol. For both Z_3_EV and Z_3_E(4S)V, the response to inducer is graded and the coefficient of variation in GFP intensities remains nearly unchanged in induced cells (i.e. the shape of the distribution of expression intensities remains unchanged) (Supplementary Figure S1). Altering the ligand specificity did, however, increase the leakiness of Z_3_E(4S)V over the original Z_3_EV construct in the absence of inducer during log-phase growth (Supplementary Figure S2). The leakiness was reduced by decreasing the promoter strength driving Z_3_E(4S)V, but it is not clear from our results if this problem is completely avoidable. Changing ligand specificity does not affect the time it takes to see induction of GFP (Supplementary Figure S2), and similar to β-estradiol, DHB has no measured effect on gene expression (Figure 6A and B) or growth (Figure 6C).

## DISCUSSION

The presented experiments sought to identify basic design principles for engineering Z_3_EV-responsive promoters, develop and characterize a toolkit for making their implementation straightforward and illustrate that the ligand specificity of Z_3_EV can be altered to respond to a non-hormone small molecule. As *S. cerevisiae* is important in both basic and applied research, these tools have many potential applications, such as engineering orthogonal pathways into yeast for making target molecules: the fact the Z_3_EV system is titratible should enable facile testing of the relationship between target gene expression levels and product yield. We envision that the different strength promoters developed here will enable simultaneous expression of multiple gene products to different levels in the presence of a single concentration of inducer, making them effective tools for metabolic engineering and pathway optimization.

### Finding cheaper non-hormone ligands

While inert with respect to yeast physiology, the use of hormone-based genetic switches is limited to academic or small- to moderate-scale experiments due to their potential for negatively impacting the environment. Only a handful of mutations are required for making a Z_3_EV variant that is strongly activated in the presence of a non-hormone small molecule, DHB; this molecule, however, is quite expensive, costing ∼$2000/g to synthesize commercially. A less soluble, cheaper DHB-like molecule in which the hydroxyl groups are replaced with methyl groups failed to induce Z_3_E(4S)V (data not shown). To make Z_3_EV a system that can be used at industrial scales, variants need to be engineered that respond not just to any non-hormone small molecules, but to ones that are (i) cheap to synthesize in large quantities and (ii) retain high specificity for the mutant receptor. Such mutant receptors may be identifiable using the previously published yeast two-hybrid approach (34), or perhaps a new selection scheme using the Z_3_EV system.

### Increasing sequence diversity

DNA assembly methods facilitate stitching large numbers of individual DNA ‘parts’ to form larger DNA assemblies. Generating these assemblies through gap-repair cloning in yeast (35) can facilitate engineering biochemical pathways (36) as well as entire genomes (37) on a single plasmid. However, all DNA assembly methods require sufficient DNA sequence diversity for parts to be properly assembled (large stretches of degenerate sequences increases the number of statistically likely assemblies).

In this manuscript, we identified several different promoter chassis that can be made inducible. Therefore, if one wants to assemble a pathway in which each gene is inducible to different levels (and is expressed at different basal levels), the promoters we identified can be readily used. In the case where more than a handful of genes require unique basal/induced expression levels, it may be necessary to find additional promoter chassis, whose basal expression can be predicted via publicly available gene expression microarray data sets. Promoters that are repressed during asexual growth (such as *HOP1* and *HOP2*) may not become inducible simply by adding Zif268 binding sites, which we anticipate is due to the presence of strong repressor elements; however, we have not completely ruled out the possibility that such promoters could become inducible with (i) additional Zif268 binding sites and/or (ii) Zif268 binding sites in different locations.

### Conclusion

We have presented synthetic biology tools that enable dynamic perturbation of gene expression levels in *S. cerevisiae* using the ATF Z_3_EV. We demonstrate that the ligand specificity of Z_3_EV can be readily re-programmed using just a handful of amino acid substitutions in the hER domain. In contrast to other published methods, these tools can be immediately applied to cells growing under diverse physiological conditions without otherwise perturbing the cellular physiology.

## SUPPLEMENTARY DATA

Supplementary Data are available at NAR Online.

Supplementary Data
