# Supplementary material for: Synthetic biology tools for programming gene expression without nutritional perturbations in Saccharomyces cerevisiae
Source: Nucleic Acids Res. 2014 Jan 20;42(6):e48. doi: 10.1093/nar/gkt1402 (PMC3973312; doi:10.1093/nar/gkt1402)
Supplement: Supplementary Data [file supp_42_6_e48__index.html]

Synthetic biology tools for programming gene expression without nutritional perturbations in Saccharomyces cerevisiae — Synthetic biology tools for programming gene expression without nutritional perturbations in Saccharomyces cerevisiae — Supplementary Data 

# Synthetic biology tools for programming gene expression without nutritional perturbations in *Saccharomyces cerevisiae*

## Supplementary Data

files

**Files in this Data Supplement:**

- Supplementary Data - pdf file
