# Supplementary material for: Synthetic biology tools for programming gene expression without nutritional perturbations in Saccharomyces cerevisiae
Source: Nucleic Acids Res. 2014 Jan 20;42(6):e48. doi: 10.1093/nar/gkt1402 (PMC3973312; doi:10.1093/nar/gkt1402)
Supplement: Supplementary Data [file supp_gkt1402_nar-03167-met-g-2013-File007.pdf]

Supplementary Information

Synthetic Biology Tools for Programming Gene Expression Without Nutritional Perturbations in *Saccharomyces cerevisiae*

R. Scott McIsaac[1,2,4,*], Patrick A. Gibney[1], Sunil S. Chandran[2], Kirsten Benjamin[2], David Botstein[1,3,*]
[1]The Lewis-Sigler Institute for Integrative Genomics, Princeton University, Princeton, NJ 08544, USA.
[2]Amyris, Inc, Emeryville, CA 94608, USA
[3]Department of Molecular Biology, Princeton University, Princeton, NJ 08544, USA.
[4]Division of Chemistry and Chemical Engineering, California Institute of Technology, Pasadena, CA 91125, USA.
*To whom correspondence should be addressed:
r.scott.mcisaac@gmail.com, botstein@genomics.princeton.edu

Sequences of promoters for Z_3EV targeting used in main text with Zif268 binding elements highlighted:

>P1
ttatattgaattttcaaaaattcttacttttttttttggatggacgcaaagaagtttaataatcatattacatggcattaccaccatata
catatccatatctaatcttacttatatgttgtggaaatgtaaagagccccattatcttagcctaaaaaaaccttctctttggaactt
tcagtaatacgcttaactgctcattgctatattgaagtgcggccgcgtgggcgtgcgtgggcgggcgtgggcgtgcgtggg
cgggcgtgggcgtgcgtgggcgtctagaccgtgcgtcctcgtcttcaccggtcgcgttcctgaaacgcagatgtgcctcgc
gccgcactgctccgaacaataaagattctacaatactagcttttatggttatgaagaggaaaaattggcagtaacctggcc
ccacaaaccttcaaattaacgaatcaaattaacaaccataggatgataatgcgattagttttttagccttatttctggggtaatt
aatcagcgaagcgatgatttttgatctattaacagatatataaatggaaaagctgcataaccactttaactaatactttcaaca
ttttcagtttgtattacttcttattcaaatgtcataaaagtatcaacaaaaaattgttaatatacctctatactttaacgtcaaggag
aaaaaactata

>P2
gcgtgggcgccaattggtgcgtgggcgccaattggtgcgtgggcgccaattggtgcgtgggcgccaattggtgcgtgggc
gccgtgcgtcctcgtcttcaccggtcgcgttcctgaaacgcagatgtgcctaacaataaagattctacaatactagcttttatg
gttatgaagaggaaaaattggcagtaacctggccccacaaaccttcaaattaacgaatcaaattaacaaccataggatg
ataatgcgattagttttttagccttatttctggggtaattaatcagcgaagcgatgatttttgatctattaacagatatataaatgga
aaagctgcataaccactttaactaatactttcaacattttcagtttgtattacttcttattcaaatgtcataaaagtatcaacaaaa
aattgttaatatacctctatactttaacgtcaaggagaaaaaactata

>P3
ttatattgaattttcaaaaattcttacttttttttttggatggacgcaaagaagtttaataatcatattacatggcattaccaccatata
catatccatatctaatcttacttatatgttgtggaaatgtaaagagccccattatcttagcctaaaaaaacctgcgtgggcgtct
ctttggaactttcagtaatacgcttgcgtgggcgaactgctcattgctatattgaagtccgtgcgtcctcgtcttcaccggtcgc
gttcctgaaacgcagatgtgcctaacaataaagattctagcgtgggcgcaatactagcttttatggttatgagcgtgggcga
gaggaaaaattggcagtaaccgcgtgggcgtggccccacaaaccttcaaattaacgaatcaaattaacgcgtgggcga
accataggatgataatgcgattagttttttagccttatttctggggtaattaatcagcgaagcgatgatttttgatctattaacaga
tatataaatggaaaagctgcataaccactttaactaatactttcaacattttcagtttgtattacttcttattcaaatgtcataaaag
tatcaacaaaaaattgttaatatacctctatactttaacgtcaaggagaaaaaactata

>P4
ttatattgaattttcaaaaattcttacttttttttttggatggacgcaaagaagtttaataatcatattacatggcattaccaccatata
catatccatatctaatcttacttatatgttgtggaaatgtaaagagccccattatcttagcctaaaaaaaccttctctttggaactt
tcagtaatacgcttaactgctcattgctatattgaagtccgtgcgtcctcgtcttcaccggtcgcgttcctgaaacgcagatgtg
cctaacaataaagattctacaatactagcttttatggttatgaagaggaaaaattggcagtaacctggccccacaaaccttc
aaattaacgaatcaaattaagcggccgcgtgggcgtgcgtgggcgggcgtgggcgtgcgtgggcgggcgtgggcgtgc
gtgggcgtctagacaaccataggatgataatgcgattagttttttagccttatttctggggtaattaatcagcgaagcgatgatt
tttgatctattaacagatatataaatggaaaagctgcataaccactttaactaatactttcaacattttcagtttgtattacttcttatt
caaatgtcataaaagtatcaacaaaaaattgttaatatacctctatactttaacgtcaaggagaaaaaactata

>P5
ttatattgaattttcaaaaattcttacttttttttttggatggacgcaaagaagtttaataatcatattacatggcattaccaccatata
catatccatatctaatcttacttatatgttgtggaaatgtaaagagccccattatcttagcctaaaaaaaccttctctttggaactt
tcagtaatacgcttaactgctcattgctatattgaagtgcggccgcgtgggcgtgcgtgggcgggcgtgggcgtgcgtggg

cgggcgtgggcgtgcgtgggcgtctagaccgtgcgtcctcgtcttcaccggtcgcgttcctgaaacgcagatgtgcctaac
aataaagattctacaatactagcttttatggttatgaagaggaaaaattggcagtaacctggccccacaaaccttcaaatta
acgaatcaaattaacaaccataggatgataatgcgattagttttttagccttatttctggggtaattaatcagcgaagcgatga
tttttgatctattaacagatatataaatggaaaagctataatagatattataacattttagtacataaaagaggcccgagaaa
atagattttaactggaataattttttgcaag

>P6
ttatattgaattttcaaaaattcttacttttttttggatggacgcaaagaagtttaataatcatattacatggcattaccaccatata
catatccatatctaatcttacttatatgttgtggaaatgtaaagagccccattatcttagcctaaaaaaccttctctttggaactt
tcagtaatacgcttaactgctcattgctatattgaagtgcggccgcgtgggcgtgcgtgggcgggcgtgggcgtgcgtggg
cgggcgtgggcgtgcgtgggcgtctagaccgtgcgtcctcgtcttcaccggtcgcgttcctgaaacgcagatgtgcctaac
aataaagattctacaatactagcttttatggttatgaagaggaaaaattggcagtaacctggccccacaaaccttcaaatta
acgaatcaaattaacaaccataggatgataatgcgattagttttttagccttatttctggggtaattaatcagcgaagcgatga
tttttgatctattaacagatatataaatggaaaagctgccgaccagcgtatacaatctcgatagttggtttcccgttctttccactc
ccgtc

>P7
gcgtgggcgtcgagcagatccgccaggcgtgtatatatagcgtggatggccaggcaactttagtgctgacacatacaggc
atatatatatgtgtgcgacgacacatgatcatatggcatgcatgtgctctgtatgtatataaaactcttgttttcttcttttctctaaat
attctttccttatacattaggacctttgcagcataaattactatacttctatagacacacaaacacaaatacacacactaaatta
ata

>P8
gcgtgggcgaattggtgcgtgggcgccaattggtgcgtgggcgtcgagcagatccgccaggcgtgtatatatagcgtgga
tggccaggcaactttagtgctgacacatacaggcatatatatatgtgtgcgacgacacatgatcatatggcatgcatgtgct
ctgtatgtatataaaactcttgttttcttcttttctctaaatattctttccttatacattaggacctttgcagcataaattactatacttcta
tagacacacaaacacaaatacacacactaaattaata

>P9
gcgtgggcgccaattggtgcgtgggcgccaattggtgcgtgggcgccaattggtgcgtgggcgtcgagcagatccgcca
ggcgtgtatatatagcgtggatggccaggcaactttagtgctgacacatacaggcatatatatatgtgtgcgacgacacatg
atcatatggcatgcatgtgctctgtatgtatataaaactcttgttttcttcttttctctaaatattctttccttatacattaggacctttgc
agcataaattactatacttctatagacacacaaacacaaatacacacactaaattaata

>P10
gcgtgggcgaattggtgcgtgggcgccaattggtgcgtgggcgaattggtgcgtgggcgccaattggtgcgtgggcgcca
attggtgcgtgggcgccaattggtgcgtgggcgccaattggtgcgtgggcgtcgagcagatccgccaggcgtgtatatata
gcgtggatggccaggcaactttagtgctgacacatacaggcatatatatatgtgtgcgacgacacatgatcatatggcatg
catgtgctctgtatgtatataaaactcttgttttcttcttttctctaaatattctttccttatacattaggacctttgcagcataaattact
atacttctatagacacacaaacacaaatacacacactaaattaata

>P11
agacgtgatttacaagacatatacaggacccaaagaaatggatttcactcatgattatttccattgaaaatcagatgattggc
ctttcattttcttcttggcggccgcgtgggcgtgcgtgggcgtctagaaaccaactgaattttgaatgattgccccccctcaattta
aagcgccaggttatcaaaactttcaacacacgctagtctgttgaaagtaaagggaagatctacgtgtgaagtgatatatgtt
tttaacctgggcggctaaattgtactttatatatagttgaaacaacagagaaagggcaggataaagaaagaaattgactgc

agtttcctatgatcatatttcatttttgtaattaacaatataatataatacacttataaacggtttaaaaacagctttatctcagaaa
agtcaggaatt

>P12
taaataacttgtagtcggatcgtacaatggctctatataggtttggaccggtatgtaacctaatatggctgactcaaaacctgc
tagatagttatccaaggcggccgcgtgggcgtgcgtgggcgggcgtgggcgtgcgtgggcgtctagatgaatttgagtctc
gtatcctttatcacatttttacaatagtggctagctatctttttcatgttaggctcacccggtattctcatgcaagatcaaacaaaa
aatggtaaagttttggactttacattgcttacagttgctttttatgaagattcaaaagattatgtcctcggcggctaaattttccatt
aataatattatcctttttacgcgaccgaaaaagagaagcgttttaagagttgtttctaacaatattaataataaagctaacgtg
aactgaccaggctctatttgaaagat

>P13
tatggccaggaaaatacataaggtttcgccgaacgacggggtcaattcgtcctttttgtacacatcgtttaatttatgaggaaa
aattgatgaatgtatcgcggccgcgtgggcgtgcgtgggcgggcgtgggcgtgcgtgggcgggcgtgggcgtgcgtggg
cgtctagactccgtagacgctcctctgaaaagtttcatgtttcctgcgcgttcctttgataggcaataaaacaatacaacgcgt
gcctttgaaaatgccaagatctatacgaggcctctaacaaaacatcgttcaggaacagagaatgctagaaatgcaaaag
ggtccctgggtactcattgaatagaaatgattgaaaatactgcgtataaaatagcacgactaaatgatactatttttatgtcga
cacggtactatttcttctttttcagataaaagtgtagcatactaaatatataccccaagta

>P14
gtgtgagacgacatcatcagacatgattcagagtgacagtattgatgtaatcaatttcctacctgaatctaaaattcccggga
gcaagatcaagatgtgtgcacctacctgtcacgcgtgggcgtaagcgggggcgggcgtgggcgatagcgggggcggt
gcgtgggcgatcgcgtgggcgtctagaatagatcaagactgcgagtgagcactcctcgagcagatccgccaggcgtgta
tatatagcgtggatggccaggcaactttagtgctgacacatacaggcatatatatatgtgtgcgacgacacatgatcatatg
gcatgcatgtgctctgtatgtatataaaactcttgttttcttcttttctctaaatattctttccttatacattaggacctttgcagcataa
attactatacttctatagacacacaaac

>P15
acggccggccaagcacgcggggataatgaactagatttccgtgtgagacgacatcgtcgatactgatgtaatcaagttcct
acctgaatctaagattcccgggagcaagatcaagatgttttcacacgatggcagaagcggccgcgtgggcgtagcggg
ggcgaagcgtgggcgtcgcgggggcgatgcgtgggcgtagcgtgggcgtctagagaccaacaggatcaagccggca
atcctcgagcagatccgccaggcgtgtatatatagcgtggatggccaggcaactttagtgctgacacatacaggcatatat
atatgtgtgcgacgacacatgatcatatggcatgcatgtgctctgtatgtatataaaactcttgttttcttcttttctctaaatattcttt
ccttatacattaggacctttgcagcataaattactatacttctatagacacacaaac

Sequences of Artificial Transcription Factors (ATFs) along with promoter and terminator elements:

>ACT1pr-Z_3EV-ENO2term

gcctctaccttgcagacccatataatataataactaaataagtaaataagacacacgcgagaacatatatacacaattac
agtaacaataacaagaggacagatactaccaaaatgtgtggggaagcgggtaagctgccacagcaattaatgcacaa
catttaacctacattcttccttatcggatcctcaaaacccttaaaaacatatgcctcaccctaacatattttccaattaaccctca
atatttctctgtcacccggcctctattttccattttcttctttacccgccacgcgtttttttctttcaaatttttttcttccttcttctttttcttcca
cgtcctcttgcataaataaataaaccgttttgaaaccaaactcgcctctctctctcctttttgaaatatttttgggtttgtttgatccttt

ccttcccaatctctcttgtttaatatatattcatttatatcacgctctcttttatcttcctttttttcctctctcttgtattcttccttcccctttct
actcaaaccaagaagaaaaagaaaaggtcaatctttgttaaagaataggatcttctactacatcagcttttagatttttcacg
cttactgcttttttcttcccaagatcgaaaatttactgaattaacagggcccccccctcgaggtcgacggtatcgataagcttga
agcaagcctcctgaaagatgggtacccgcccatatgcttgccctgtcgagtcctgcgatcgccgcttttctcgctcggatga
gcttacccgccatatccgcatccataccggtcagaagcccttccagtgtcgaatctgcatgcgtaacttcagtcgtagtgac
caccttaccacccacatccgcacccacacaggcgagaagccttttgcctgtgacatttgtgggaggaagtttgccaggagt
gatgaacgcaagaggcataccaaaatccatacaggtggcggaggcacacctgcagctgcgtcgactctagaggatcc
atctgctggagacatgagagctgccaacctttggccaagcccgctcatgatcaaacgctctaagaagaacagcctggcct
tgtccctgacggccgaccagatggtcagtgccttgttggatgctgagccccccatactctattccgagtatgatcctaccaga
cccttcagtgaagcttcgatgatgggcttactgaccaacctggcagacagggagctggttcacatgatcaactgggcgaa
gagggtgccaggctttgtggatttgaccctccatgatcaggtccaccttctagaatgtgcctggctagagatcctgatgattgg
tctcgtctggcgctccatggagcacccagtgaagctactgtttgctcctaacttgctcttggacaggaaccagggaaaatgt
gtagagggcatggtggagatcttcgacatgctgctggctacatcatctcggttccgcatgatgaatctgcagggagaggag
tttgtgtgcctcaaatctattattttgcttaattctggagtgtacacatttctgtccagcaccctgaagtctctggaagagaagga
ccatatccaccgagtcctggacaagatcacagacactttgatccacctgatggccaaggcaggcctgaccctgcagcag
cagcaccagcggctggcccagctcctcctcatcctctcccacatcaggcacatgagtaacaaaggcatggagcatctgta
cagcatgaagtgcaagaacgtggtgcccctctatgacctgctgctggagatgctggacgcccaccgcctacatgcgccc
actagccgtggaggggcatccgtggaggagacggaccaaagccacttggccactgcgggctctacttcatcggagctcc
acttagacggcgaggacgtggcgatggcgcatgccgacgcgctagacgatttcgatctggacatgttgggggacgggg
attccccgggtccgggatttacccccacgactccgcccctacggcgctctggatatggccgacttcgagtttgagcagat
gtttaccgatgcccttggaattgacgagtacggtgggtagatccccgcgtgcttggccggccgtagtgcttttaactaagaatt
attagtcttttctgcttatttttcatcatagtttagaacactttatattaacgaatagtttatgaatctatttaggtttaaaaattgatac
agttttataagttactttttcaaagactcgtgctgtctattgcataatgcactggaaggggaaaaaaaaggtgcacacgcgtg
gctttttcttgaatttgcagtttgaaaaataactacatggatgataagaaaacatggagtacagtcactttgagaaccttcaat
cagctggtaacgtcttcgttaattggatactcaaaaaagatggatagcatgaatcacaagatggaaggaaatgcgggcc
acgaccacagtgatatgcatatgggagatggagatgatacctgttcgatgaatatgctattttcgtggtcatacaagaatac
gtgtgtcgtctttgaatggtggcatatcaagaccctgcctggactgat

> CYC1pr-Z_3(4S)EV-YPR052Cterm

ttggaaaaccaagaaatgaattatatttccgtgtgagacgacatcgtcgaatatgattcagggtaacagtattgatgtaatca
atttcctacctgaatctaaaattcccgggagcaagatcaagatgttttcaccgatctttccggtctctttggccggggtttacgg
acgatggcagaagaccaaagcgccagttcatttggcgagcgttggttggtggatcaagcccacgcgtaggcaatcctcg
agcagatccgccaggcgtgtatatatagcgtggatggccaggcaactttagtgctgacacatacaggcatatatatatgtgt
gcgacaacacatgatcatatggcatgcatgtgctctgtatgtatataaaactcttgttttcttcttttctctaaatattctttccttatac
attaggacctttgcagcataaattactatacttctatagacacacaaacacaaatacacacactaaattaataacctcccgc
gacctccaaaatcgaactaccttcacaatgggtactagaccatacgcttgtccagttgagtcttgtgacagaagattctctag
atccgacgaattaacccgtcacattagaattcatactggtcaaaaacctttccaatgtagaatctgcatgagaaacttttctcg
ttctgatcacttgactactcatatcagaacccacactggtgaaaaacctttcgcttgtgatatttgtggtagaaaattcgccaga
tctgatgaaagaaagcgtcacactaaaatccacactggtggtggtggtactccagctgctgcttccaccttggaagaccca
tctgctggtgacatgcgtgctgctaacttgtggccttctccattaatgatcaaaagatctaaaaagaattctttggctttgtctttg
actgctgatcaaatggtttctgctttattagacgccgaaccaccaattttatactctgaatatgatcctactcgtccattttccgaa
gcctctatgatgggtttaattaccaacttgatggatagagaattagtccatatgattaactgggccaagcgtgtcccaggttttg
ttgatttaaccttacacgatcaagttcatttgttggaatgtgcttggttggaaatcttgcaaattggtttggtttggagatccatgga
acatccagttaaattattgttcgctcctaatttgttgttggacagaaaccaaggtaagtgtgttgaaggtatggttgaaattttcg
atatgttgttggctacttcttctagattcagaatgatgaacttgcaaggtgaggaattcgtttgtttgaaatccattatcttgttgaat

tctggtgtttacactttcttgtcttccactttaaagtctttggaagaaaaggaccacatccatagagtcttggacaagatcaccg
acaccttaattcatttaatggccaaggctggtttaaccttgcaacaacaacaccaacgtttggcccaattgttgttgatcttgtct
cacattcgtcatatgtctaataaatctatggaacacttggactccatgaagtgtaagaacgtcgtcccattgtacgacttattgt
tagaaatgttggacgctcatagattacatgccccaacttctagaggtggtgcctccgtcgaggaaactgatcaatcccatttg
gctactgctggttccacttcttccgaattacatttagatggtgaggatgtcgctatggcccacgctgatgctttggacgatttcga
tttggacatgttgggtgacggtgactccccaggtccaggtttcaccccacatgattccgccccatacggtgctttggatatggc
cgactttgaattcgaacaaatgtttactgatgctttgggtatcgacgaatatggtggttagggatacttcctccccgcgtttttgtc
tgtgtgatttaatttcttttcctcttgttctttttgaaacttgctttattatctctctcctttctattcctcttttctctactgccctttttctatatcta
tacatatgattaaataaataaataaaaaaattgtattattaatatatattttatcatcatcgag

> ACT1pr-Z_3(4S)EV-YPR052Cterm

acacaattacagtaacaataacaagaggacagatactaccaaaatgtgtggggaagcgggtaagctgccacagcaatt
aatgcacaacatttaacctacattcttccttatcggatcctcaaaacccttaaaaacatatgcctcaccctaacatattttccaa
ttaaccctcaatatttctctgtcacccggcctctattttccattttcttctttacccgccacgcgtttttttctttcaattttttttcttctttctt
ctttttcttccacgtcctcttgcataaataaataaaccgttttgaaaccaaactcgcctctctctctcctttttgaaatatttttgggttt
gtttgatcctttccttcccaatctctcttgtttaatatatattcatttatatcacgctctcttttttatcttccttttttttcctctctcttgtattcttc
cttcccctttctactcaaaccaagaagaaaaagaaaaggtcaatctttgttaaagaataggatcttctactacatcagcttta
gatttttcacgcttactgctttttttcttcccaaacctcccgcgacctccaaaatcgaactaccttcacaatgggtactagaccat
acgcttgtccagttgagtcttgtgacagaagattctctagatccgacgaattaacccgtcacattagaattcatactggtcaaa
aacctttccaatgtagaatctgcatgagaaacttttctcgttctgatcacttgactactcatatcagaacccacactggtgaaa
aacctttcgcttgtgatatttgtggtagaaaattcgccagatctgatgaaagaaagcgtcacactaaaatccacactggtggt
ggtggtactccagctgctgcttccaccttggaagacccatctgctggtgacatgcgtgctgctaacttgtggccttctccattaa
tgatcaaaagatctaaaaagaattctttggctttgtctttgactgctgatcaaatggtttctgctttattagacgccgaaccacca
attttatactctgaatatgatcctactcgtccattttccgaagcctctatgatgggtttaattaccaacttgatggatagagaatta
gtccatatgattaactgggccaagcgtgtcccaggttttgttgatttaaccttacacgatcaagttcatttgttggaatgtgcttgg
ttggaaatcttgcaaattggtttggtttggagatccatggaacatccagttaaattattgttcgctcctaatttgttgttggacaga
aaccaaggtaagtgtgttgaaggtatggttgaaattttcgatatgttgttggctacttcttctagattcagaatgatgaacttgca
aggtgaggaattcgtttgtttgaaatccattatcttgttgaattctggtgtttacactttcttgtcttccactttaaagtctttggaaga
aaaggaccacatccatagagtcttggacaagatcaccgacaccttaattcatttaatggccaaggctggtttaaccttgcaa
caacaacaccaacgtttggcccaattgttgttgatcttgtctcacattcgtcatatgtctaataaatctatggaacacttggactc
catgaagtgtaagaacgtcgtcccattgtacgacttattgttagaaatgttggacgctcatagattacatgccccaacttctag
aggtggtgcctccgtcgaggaaactgatcaatcccatttggctactgctggttccacttcttccgaattacatttagatggtgag
gatgtcgctatggcccacgctgatgctttggacgatttcgatttggacatgttgggtgacggtgactccccaggtccaggtttc
accccacatgattccgccccatacggtgctttggatatggccgactttgaattcgaacaaatgtttactgatgctttgggtatcg
acgaatatggtggttagggatacttcctccccgcgtttttgtctgtgtgatttaatttcttttcctcttgttctttttgaaacttgctttatta
tctctctcctttctattcctcttttctctactgccctttttctatatctatacatatgattaaataaataaataaaaaaattgtattattaat
atatattttatcatcatcgag

Sequence of cassette used for selective integration:

>URA3
acgaaaatcgttattgtcttgaaggtgaaatttctactcttattaatggtgaacgttaagctgatgctatgatggaagctgattgg
tcttaacttgcttgtcatcttgctaatggtcattggctcgtgttattacttaagttatttgtactcgttttgaacgtaatgctaatgatcat
cttatggaataatagtgagtggtttcagggtccataaagcttttcaattcatcttttttttttttgttcttttttttgattccggtttctttgaa
atttttttgattcggtaatctccgagcagaaggaagaacgaaggaaggagcacagacttagattggtatatatacgcatatg

tggtgttgaagaaacatgaaattgcccagtattcttaacccaactgcacagaacaaaaacctgcaggaaacgaagataa
atcatgtcgaaagctacatataaggaacgtgctgctactcatcctagtcctgttgctgccaagctatttaatatcatgcacgaa
aagcaaacaaacttgtgtgcttcattggatgttcgtaccaccaaggaattactggagttagttgaagcattaggtcccaaaat
ttgtttactaaaaacacatgtggatatcttgactgatttttccatggagggcacagttaagccgctaaaggcattatccgccaa
gtacaatttttactcttcgaagacagaaaatttgctgacattggtaatacagtcaaattgcagtactctgcgggtgtatacaga
atagcagaatgggcagacattacgaatgcacacggtgtggtgggcccaggtattgttagcggtttgaagcaggcggcgg
aagaagtaacaaaggaacctagaggccttttgatgttagcagaattgtcatgcaagggctccctagctactggagaatata
ctaagggtactgttgacattgcgaagagtgacaaagattttgttatcggctttattgctcaaagagacatgggtggaagagat
gaaggttacgattggttgattatgacacccggtgtgggtttagatgacaagggagacgcattgggtcaacagtatagaacc
gtggatgatgtggtctctacaggatctgacattattattgttggaagaggactatttgcaaagggaagggatgctaaggtag
agggtgaacgttacagaaaagcaggctgggaagcatatttgagaagatgcggccagcaaaactaaaaaactgtattat
aagtaaatgcatgtatactaaactcacaaattagagcttcaatttaattatatcagttattaccacgaaaatcgttattgtcttga
aggtgaaatttctactcttattaatggtgaacgttaagctgatgctatgatggaagctgattggtcttaacttgcttgtcatcttgct
aatggtcatatggctcgtgttattacttaagttatttgtactcgttttgaacgtaatgctaatgatcatcttatggaataatagtga

Sequence of GFP reporter:

>GFP(Dasher)-Gal80term
atgacagctttaactgaaggggccaagcttttcgaaaaagagatcccatatatcaccgaacttgaaggtgacgttgagggt
atgaagtttatcataaaaggagaaggcacaggtgatgcgacaacgggtacaatcaaggcaaagtacatttgtacaactg
gggacctgcctgtcccatgggccactttggtgtctactttgtcttacggcgtacaatgttttgctaagtacccttcacacatcaaa
gatttctttaagtctgcaatgcctgaaggatacacacaggaacgtacaatttcatttgagggcgacggtgtctataaaacaa
gagctatggttacttatgaaagaggttccatctacaacagagtgacactaacgggcgaaaatttcaaaaaggatggacat
attttgcgtaaaaacgtagctttccaatgcccaccatcaatactatacattctgccagatactgtaaacaatggtattagagtc
gagtttaatcaagcttatgatatagaaggtgtcactgaaaaattggttacaaaatgcagccaaatgaatagaccattggca
ggatctgccgctgtgcatatccctagataccatcacattacctaccacaccaaattaagtaaagacagggatgaacgaag
agatcatatgtgtttagttgaggttgttaaggcagttgatctcgacacttaccaataaaagcatcttgccctgtgcttggccccc
agtgcagcgaacgttataaaaacgaatactgagtatatatctatgtaaaacaaccatatcatttcttgttctgaactttgtttacc
taactagttttaaatttccctttttcgtgcatgcgggtgttcttatttattagcatactacatttgaaatatcaaatttccttagtagaaa
agtgagagaaggtgcactgacaca

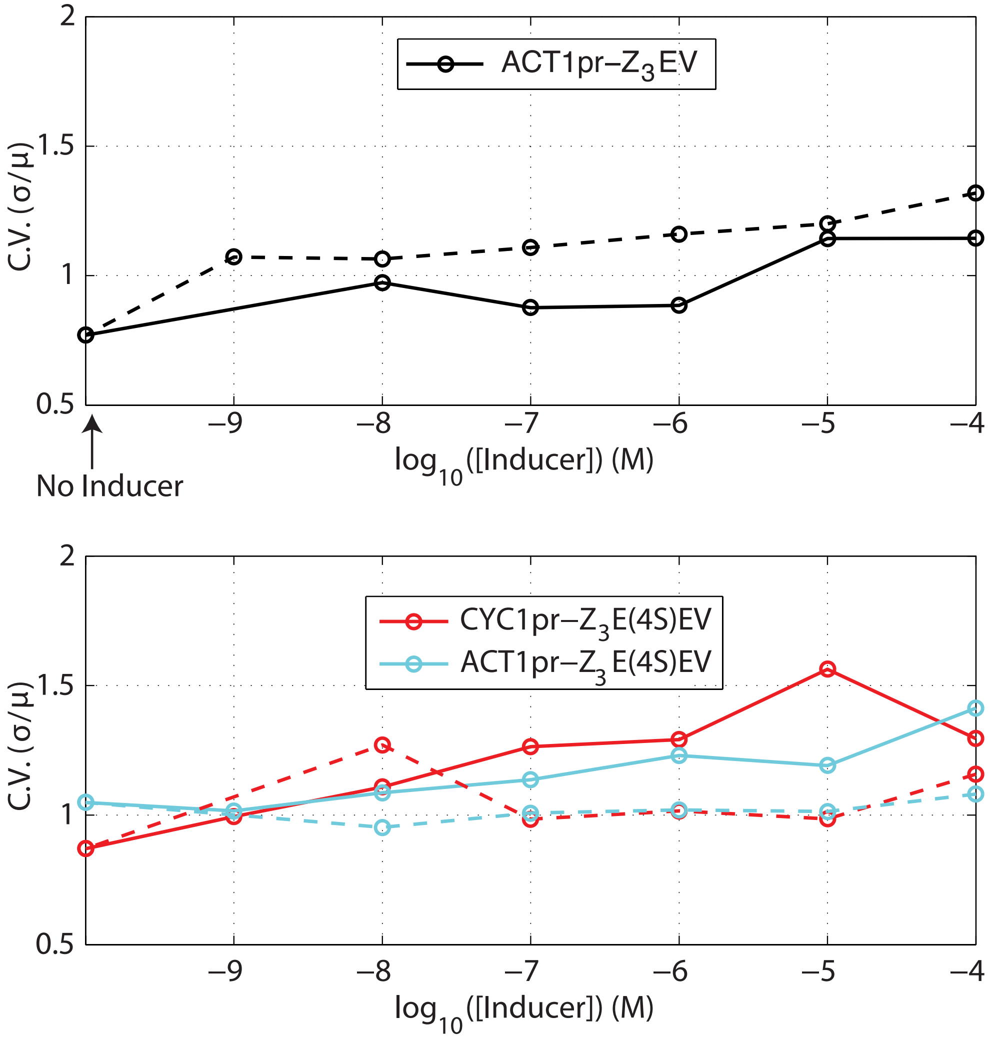

Figure S1: The coefficient of variation (C.V.) of GFP expression in a strain containing Z_3EV (top) or $Z_3E(4S)V$ (bottom) in the presence of β-estradiol (solid lines) or DHB (dashed line).

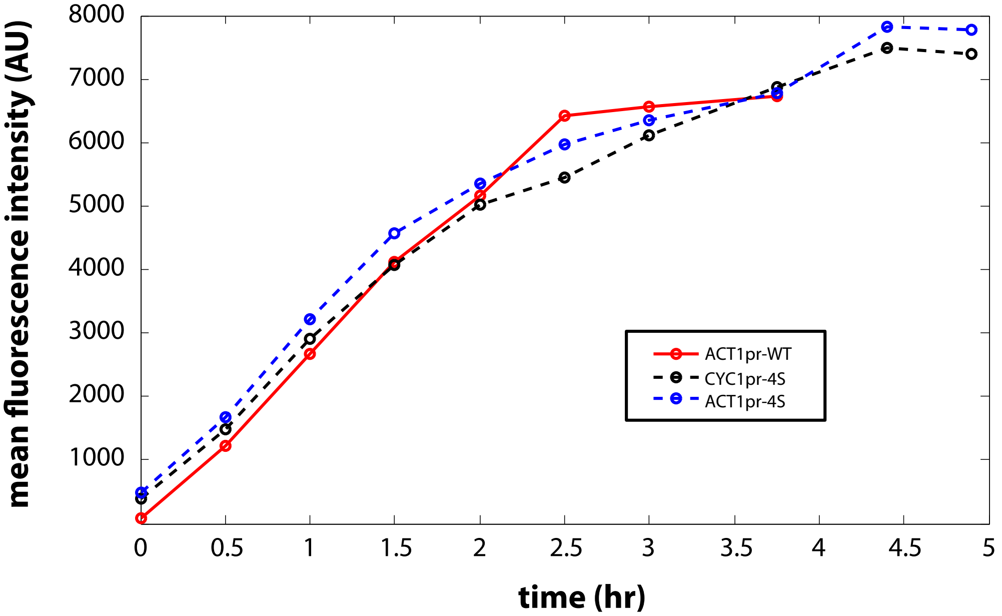

Figure S2: Induction of GFP by ACT1pr-Z_3EV (red), CYC1pr- Z_3E(4S)V (black), or ACT1pr- Z_3E(4S)V as function of time. ACT1pr-Z_3EV was induced with 1 μM β-estradiol. CYC1pr- Z_3E(4S)V and ACT1pr- Z_3E(4S)V were induced with 1 μM DHB.
